# Beneficial effects of combined resveratrol and metformin therapy in treating diet‐induced insulin resistance

**DOI:** 10.14814/phy2.12877

**Published:** 2016-08-01

**Authors:** Scott Frendo‐Cumbo, Rebecca E. K. MacPherson, David C. Wright

**Affiliations:** ^1^ Department of Human Health and Nutritional SciencesUniversity of GuelphGuelphOntarioCanada

**Keywords:** Combination therapy, insulin resistance, metformin, resveratrol

## Abstract

The polyphenol compound resveratrol (RSV) has attracted attention due to its reputed beneficial effects on insulin sensitivity. Our lab has previously identified protective effects of RSV against the development of type 2 diabetes in rats. These effects occurred in a manner similar to thiazolidinedione's (TZDs), a class of insulin sensitizing drugs. TZDs are commonly prescribed in combination with metformin (MET) and thus we sought to examine the combined effects of RSV and MET in treating insulin resistance. Male C57BL6 mice were fed a low‐ (LFD; 10% Kcal from fat) or high‐fat diet (HFD; 60% Kcal from fat) for 9 weeks to induce glucose and insulin intolerance. HFD mice were then assigned to control (HFD), MET (231.28 ± 12.24 mg/kg/day), RSV (93.68 ± 3.51 mg/kg/day), or combined (COM; MET 232.01 ± 17.12 mg/kg/day and RSV 92.77 ± 6.92 mg/kg/day) treatment groups. Changes in glucose and insulin tolerance and tissue‐specific insulin signaling were measured 4 weeks post‐treatment. RSV or MET alone did not have beneficial effects on glucose tolerance, although MET significantly improved insulin tolerance compared to HFD. Glucose and insulin tolerance were significantly improved in COM compared to HFD and this was mirrored by enhanced insulin‐stimulated AKT phosphorylation in triceps muscle and inguinal subcutaneous adipose tissue in COM compared to HFD mice. Improvements with COM treatment were not explained by differences in body weight, adiposity, or markers of adipose tissue inflammation. In summary, this study provides evidence of beneficial effects of combined RSV and MET therapy in treating impairments in glucose homeostasis.

## Introduction

The incidence of type 2 diabetes (T2D) is increasing at an alarming rate and is one of the leading causes of non‐communicable disease deaths in the world (Mathers and Loncar 2006). Insulin resistance is a precursor to the development of T2D and therefore it is important to identify treatment options for this condition. Two drugs that are commonly used in the management of perturbed glucose metabolism are metformin (MET) and thiazolidinediones (TZDs). MET reduces hepatic liver glucose output (Hundal et al. 2000) and enhances skeletal muscle glucose uptake (Musi et al. 2002) and insulin signaling (Luna et al. 2006), whereas TZDs increase insulin sensitivity (Miyazaki et al. 2001, 2002), in part, due to reductions in adipose tissue inflammation (Xu et al. 2003) and increases in the insulin sensitizing adipokine (Yamauchi et al. 2002; Bruce et al. 2005) adiponectin (Nawrocki et al. 2006). As MET and TZDs work through distinct mechanisms, it is not surprising that dual therapy with both drugs leads to greater improvements in glucose homeostasis than either drug alone (Inzucchi et al. 1998; DeFronzo 1999; Stafford and Elasy 2007). Although effective in treating impaired glucose homeostasis, TZDs have wide ranging adverse effects including increased risk of congestive heart failure, myocardial infarction, and bladder cancer (Nissen and Wolski 2007; Turner et al. 2014). Given this, it would be advantageous to identify treatment approaches that might possess some of the same beneficial effects as TZDs.

The polyphenol compound resveratrol (RSV) has gained attention for its ability to protect against the development of insulin resistance (Baur et al. 2006; Lagouge et al. 2006; Beaudoin et al. 2013). For example, we have recently shown that dietary RSV supplementation prevents the development of T2D in Zucker Diabetic Fatty rats and this was associated with increases in serum adiponectin levels and adipose tissue mitochondrial content and respiration (Beaudoin et al. 2013), effects similar to those of TZDs (Koh et al. 2007). In contrast to TZDs, RSV possesses cardioprotective effects (Raj et al. 2014; Sung and Dyck 2015) and has been reported to attenuate growth rates of bladder cancer cells (Wu et al. 2014). Given these points, it seems reasonable to assume that RSV could be an attractive therapeutic option, either alone, or in combination with MET, in treating insulin resistance. Surprisingly, to the best of our knowledge, this has never been explored. Within this context, the purpose of the current investigation was to compare the effects of RSV and MET, alone and in combination, in treating glucose intolerance and insulin resistance in mice fed a high‐fat diet. We hypothesized that dual therapy would be more effective than either compound alone.

## Materials and Methods

### Materials

Trans‐resveratrol (CAT# 70675) and metformin (CAT# 13118) were purchased from Cayman Chemicals (Ann Arbor, MI). Low‐fat (LFD: 10% kcal from fat; CAT# D12450B) and high‐fat (HFD: 60% kcal from fat; CAT# D12492) diets were purchased from Research Diets (New Brunswick, NJ). Insulin was from Eli Lilly (Toronto, ON, Canada) and glucose from BioShop (Burlington, ON, Canada). Reagents, molecular weight marker and nitrocellulose membranes for SDS‐PAGE were purchased from Bio‐Rad. Antibodies against glyceraldehyde 3‐phosphate dehydrogenase (GAPDH; CAT# ab8245) and vinculin (CAT# ab129002) were obtained from Abcam (Toronto, ON, Canada). Total Akt (CAT# 4685), p‐AKT threonine 308 (CAT# 9275) and p‐AKT serine 473 (CAT# 9271) antibodies were from Cell Signaling (Danvers, MA). An antibody against the alpha subunit of glucose 6 phosphatase (G6Pase) (CAT# 25840) was purchased from Santa Cruz Biotechnology (Santa Cruz, CA). Antibodies against phosphoenolpyruvate carboxykinase (PEPCK) (CAT# 10004943) were a product of Cayman Chemicals (Ann Arbor, MI). Western‐Lightening Plus Enhanced Chemiluminescence substrate (CAT# NEL105001EA) was purchased from Perkin Elmer (Waltham, MA). Horseradish peroxidase‐conjugated donkey anti‐rabbit and goat anti‐mouse IgG secondary antibodies were from Jackson ImmunoResearch Laboratories (West Grove, PA). SuperScript II Reverse Transcriptase, random primers and dNTP were products from Invitrogen (Burlington, ON, Canada). RNeasy mini kits and Quiazol were from Qiagen (Toronto, ON, Canada). Taqman gene expression assays for mouse GAPDH (CAT# 4352932), tumor necrosis factor alpha (TNF*α*) (CAT #Mm0044358_m1), F4/80 (Mm00802529_m1), and adiponectin (CAT# Mm00456425_m1) were from Applied Biosystems. An ELISA for mouse adiponectin (CAT # EZMADP‐60K) was obtained from Millipore (Etobicoke, ON).

### Animals

Animal protocols were approved by the University of Guelph Animal Care Committee and met Canadian Council on Animal Care (CCAC) guidelines. Male C57BL6 mice, 8 weeks of age (Charles River), were individually housed with a 12:12 h light:dark cycle and ad libitum access to water and experimental diets. Mice were fed a LFD or HFD for 9 weeks, after which intraperitoneal glucose and insulin tolerance tests (IPGTT and IPITT) were performed to confirm the presence of perturbed glucose metabolism. HFD mice were randomly assigned to HFD‐control, HFD‐metformin (MET; 250 mg/kg/day), HFD‐resveratrol (RSV; 100 mg/kg/day), or a combination of both (COM; MET: 250 mg/kg/day and RSV: 100 mg/kg/day). The doses of RSV and MET used in this study are similar to those used in previous rodent‐based investigations (Fujita et al. 2002; Lagouge et al. 2006; Hou et al. 2010; Beaudoin et al. 2013; Kristensen et al. 2013). In a pilot experiment, we found that 250 mg/kg/day MET treatment lowered the glucose area under the curve (AUC), during a glucose tolerance test compared to untreated HFD mice (HFD 1941 ± 344, MET 1464 ± 121) but not to levels seen in mice fed a low‐fat diet (1000 ± 146). A lower dose of MET (62.5 mg/kg/day) had negligible effects (1821 ± 286) on HFD‐induced glucose tolerance.

Body weight (BW) and glucose homeostasis were similar at the onset of treatment. Treatments were added directly to the food, in their powdered form, and manually mixed on a weekly basis, with amounts based on average daily food intake and BW. The average dose of MET and RSV consumed were than calculated at termination, based on average BW and daily food intake per week as well as the dilution of treatment administered in food. IPGTT and IPITT were repeated following 4 weeks of treatment. Animal and food weights were monitored 1 and 3 days/week, respectively, throughout the duration of the study.

### Glucose and insulin tolerance testing

Intraperitoneal glucose and insulin tolerance tests (IPGTT and IPITT) were performed as measures of glucose homeostasis. For the IPGTT, mice were fasted for 6 h prior to an IP injection of glucose (2 g/kg BW). For the IPITT measures, food was removed immediately prior to an IP injection of insulin (0.5 U/kg). Blood glucose concentrations were determined via tail vein sampling using a hand‐held glucometer (Freestyle Lite, Abbott). IPGTT and IPITT measures were performed 48 hours apart as previously described (Beaudoin et al. 2013), with mice given ad libitum access to their respective diets between tests. Relative changes in IPGTT and IPITT measures compared to the HFD were calculated and independent MET and RSV changes were summed to predict the effects of combined therapy.

### Tissue collection

After 4 weeks of treatment, and 72 h following the last tolerance test, mice were anesthetized with sodium pentobarbital (5 mg/100 g, IP injection) and inguinal subcutaneous (scAT) and epididymal (eWAT) adipose tissue depots and triceps from the left side of mice were excised, weighed, and flash frozen or fixed in formalin. Mice were injected with insulin (1.25 U/kg) and 10 min postinjection, contralateral tissues and the liver were excised, weighed, and flash frozen. Intrathoracic blood was collected at this time. Tissues were stored at −80°C until further analysis.

### Histology

Approximately 100 mg of eWAT and scAT were fixed in 10% neutral‐buffered formalin (VWR, Mississauga, ON, Canada), dehydrated in 70% ethanol (Fisher Scientific), and embedded in paraffin. Five micrometer sections were mounted on 1.2 mm Superfrost slides, stained with Harris hematoxylin and eosin stock (H&E; Fisher Scientific) and imaged (Olympus FSX 100 light microscope, Olympus, Tokyo, Japan) as previously described (MacPherson et al. 2015). Cells were sampled in each image to determine cross‐sectional area (>200 cells/mouse) (ImageJ software, National Institute of Mental Health, Bethesda, MD). The presence of crown‐like structures was determined as we have described in detail previously (MacPherson et al. 2016).

### Western blotting

Samples were homogenized in 3 (scAT and eWAT), 20 (triceps), or 30 (liver) volumes of NP40 Cell Lysis Buffer (Life Technologies; CAT# FNN0021) supplemented with phenylmethylsulfonyl fluoride and protease inhibitor cocktail (Sigma; CAT# 78830, CAT# P2714) using a FastPrep‐24 Tissue Homogenizer (MP Biomedicals) as previously described (MacPherson et al. 2015). Homogenized samples were centrifuged at 4°C for 10 min at 1500 × *g* and the supernatant collected from liver and skeletal muscle, while the infranatant was collected from adipose tissue. A bicinchoninic acid assay was performed to determine protein content of the homogenate (Smith et al. 1985). Equal amounts of protein were then electrophoretically separated on 10% SDS‐PAGE gels and transferred to nitrocellulose membranes. Membranes were blocked for 1 h at room temperature in 5% non‐fat dry milk‐TBST (tris‐buffered saline/0.1% tween 20). Membranes were then incubated in primary antibody diluted 1:1000 in 5% BSA (Bovine Serum Albumin)‐TBST overnight with gentle agitation at 4°C. Following a 1‐hour incubation at room temperature with appropriate secondary antibodies, in 1% non‐fat dry milk‐TBST, membranes were washed and proteins visualized by Western Lightning Plus‐ECL using a Flourochem HD2 imager (Cell Biosciences) and bands quantified using Alpha Innotech software (Santa Clara, CA). A housekeeping protein (GAPDH) was measured in each gel to ensure equal loading.

### RT‐PCR

Changes in mRNA expression were determined using real‐time qPCR as described in detail previously by our laboratory (MacPherson et al. 2015). RNA was isolated from scAT and eWAT using a Qiagen RNeasy kit according to the manufacturer's instructions. Complementary DNA (cDNA) was synthesized from 1μg of total RNA using SuperScript II Reverse Transcriptase, random primers, and dNTP. Real‐time PCR was carried out using the CFX Connect Real‐Time PCR Detection System (Bio‐Rad). Each well contained 1 *μ*L of cDNA template, 8 *μ*L of RNase free water, 1 *μ*L gene expression assay, and 10 *μ*L of Taqman Fast Universal PCR Master Mix. Relative differences between groups were determined using the 2^−∆∆CT^ method (Livak and Schmittgen 2001). The amplification efficiencies of the gene of interest and the housekeeping gene were equivalent and the expression of GAPDH did not change between groups.

### Adiponectin ELISA

Circulating concentrations of adiponectin were measured by ELISA as described previously (Beaudoin et al. 2013).

### Statistical analysis

Differences between LFD and HFD before treatment were examined by one‐tailed *t*‐test. A one‐tailed *t*‐test was chosen a priori based on the prevalence of literature demonstrating impaired glucose and insulin tolerance following long‐term HFD. For examination of blood glucose differences at individual time‐points during glucose and insulin tolerance testing in LFD and HFD mice, a repeated measures ANOVA was performed with a Sidak's multiple comparisons test. A one‐way ANOVA with Tukey's post hoc analysis was used to determine differences between treatment groups. When data were not normally distributed, as measured with Shapiro–Wilk normality testing, data were log transformed to achieve normal distribution. To examine differences in AKT phosphorylation between and within groups (basal vs. insulin‐stimulated) and differences in blood glucose at individual time‐points during IPGTT and IPITT between treatment groups, a two‐way repeated measures ANOVA with Tukey's post hoc analysis was used. A relationship was considered significant when *P* < 0.05.

## Results

### High‐fat diet induces glucose and insulin intolerance

**Figure 1:**
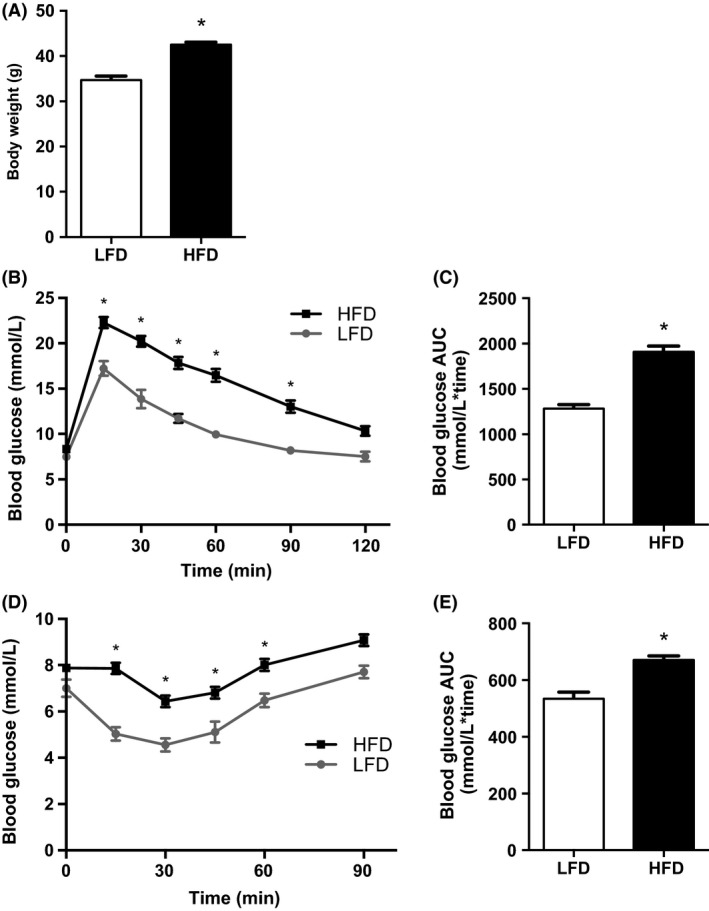
High‐fat diet (HFD) increases body weight and impairs glucose and insulin tolerance. Body weight (A), intraperitoneal glucose tolerance tests (IPGTT) (B), IPGTT area under the curve (AUC) (C), IPITT (D) and Intraperitoneal insulin tolerance tests (IPITT) AUC (E) for low‐fat diet (LFD) (*n* = 12) and HFD (*n* = 56) mice. Values are mean ± SEM. Significance *P < 0.05.

Animals fed the HFD weighed significantly more than LFD mice following 9 weeks of feeding (Fig. 1A) and displayed impairments in glucose homeostasis as evidenced by higher glucose AUCs during the IPGTT (Fig. 1B and C) and IPITT (Fig. 1D and E). Specifically, HFD mice displayed significantly increased blood glucose concentrations between 15 and 90 min during the IPGTT and between 15 and 60 min during the IPITT, which is indicative of impaired whole‐body glucose homeostasis (Fig. 1B and D). Treatment interventions (MET, RSV, and COM) were initiated at this time. As we were interested in examining the effects of RSV and/or MET in treating impaired glucose homeostasis, only HFD mice were studied. Body weights and glucose and insulin tolerance were not different between groups at the onset of treatment (data not shown).

### Food intake, body weight, and adiposity

**Figure 2:**
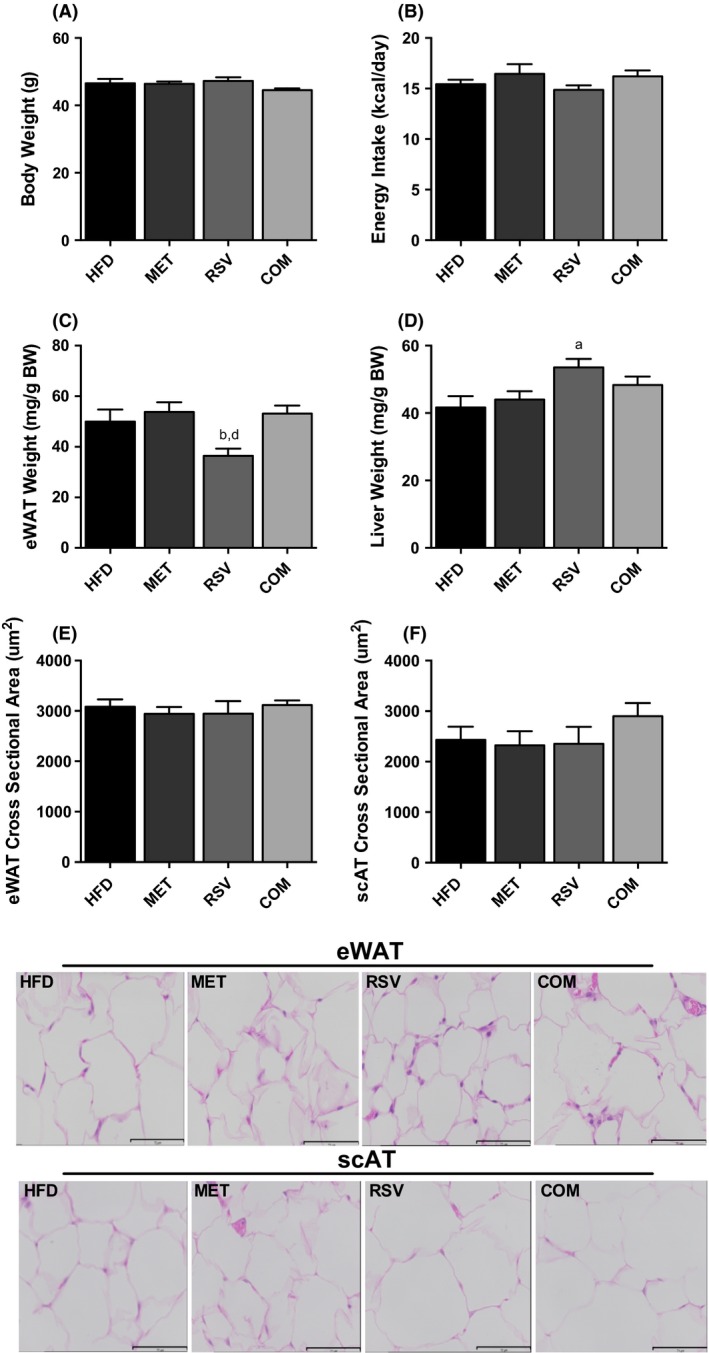
The effects of resveratrol (RSV), metformin (MET), and combined (COM) on body weight (A), food intake (B) eWAT weight (C), liver weight (D), and fat cell size (E–G). Values are presented as mean ± SEM for 9–16 mice/group in (A–D), and 5 mice/group in (E–G). Significance (*P* < 0.05) is denoted by letters: a different than HFD, b different than MET, c different than RSV and d different than COM.

Four weeks of MET (231.28 ± 12.24 mg/kg/day), RSV (93.68 ± 3.51 mg/kg/day), or COM (MET 232.01 ± 17.12 mg/kg/day; RSV 92.77 ± 6.92 mg/kg/day) treatment had no effect on BW or food intake compared to HFD (Fig. 2A and B). However, RSV exhibited significantly reduced eWAT weight compared to MET and COM (Fig. 2C), as well as significantly increased liver weight compared to HFD (Fig. 2D). No differences were observed in adipocyte size in eWAT or scAT between groups (Fig. 2E–G).

### Whole‐body glucose homeostasis and insulin action

**Figure 3:**
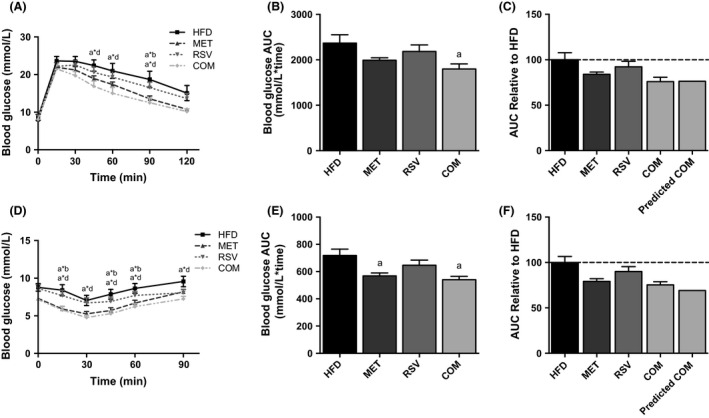
Combined (COM) therapy improves high‐fat diet (HFD)‐induced glucose (A–C) and insulin intolerance (D–F). Changes in glucose over time (A, D), the glucose AUC (B, E), and the predicted compared to actual combined effects of treatment (C, F) are shown. Data are presented as means ± SEM for 11–17 mice/group. Significance (*P* < 0.05) is denoted by letters: a different than HFD, b different than MET, c different than RSV and d different than COM. Significant differences at individual time points of the IPGTT and IPITT curves are denoted by an asterisk (*) between letters of corresponding groups.

To assess the effects of MET and RSV on glucose homeostasis, we first performed IPGTTs following 4 weeks of treatment with MET, RSV, and COM. As shown in Figure 3A and B, both RSV and MET treatment alone did not improve glucose intolerance in mice fed a HFD, although MET displayed significantly lower blood glucose than HFD at 90 min. However, when given in combination, glucose tolerance was significantly improved, as shown by a lower IPGTT AUC in COM compared to HFD. COM mice had significantly lower blood glucose compared to HFD at 45, 60, and 90 min during the IPGTT. We then calculated differences in the glucose AUCs relative to the untreated HFD mice (i.e., HFD mice = 100). Interestingly, the predicted improvement in glucose tolerance with both compounds in combination was essentially identical to the observed COM effects (Fig. 3C).

We further evaluated the individual and combined effects of MET and RSV on glucose homeostasis using an IPITT. RSV alone did not improve insulin tolerance compared to HFD (Fig. 3D and E). However, MET and COM significantly lowered the IPITT AUC compared to HFD, indicative of improved insulin tolerance in these groups (Fig. 3E). The COM group had significantly lower blood glucose compared to HFD at 15, 30, 45, 60, and 90 min, while MET was only significantly different at 15, 45, and 60 min during the IPITT. The predicted additive effects of combined treatment were again similar to the observed improvements with COM treatment (Fig. 3F).

### Tissue specific insulin action

In order to evaluate tissue‐specific effects of our treatments on insulin action, AKT phosphorylation at S473 and T308 activation sites were examined in triceps and adipose tissue prior to and following an IP bolus injection of insulin. Liver was only examined post‐insulin, as pre‐insulin samples could not be obtained.

**Figure 4:**
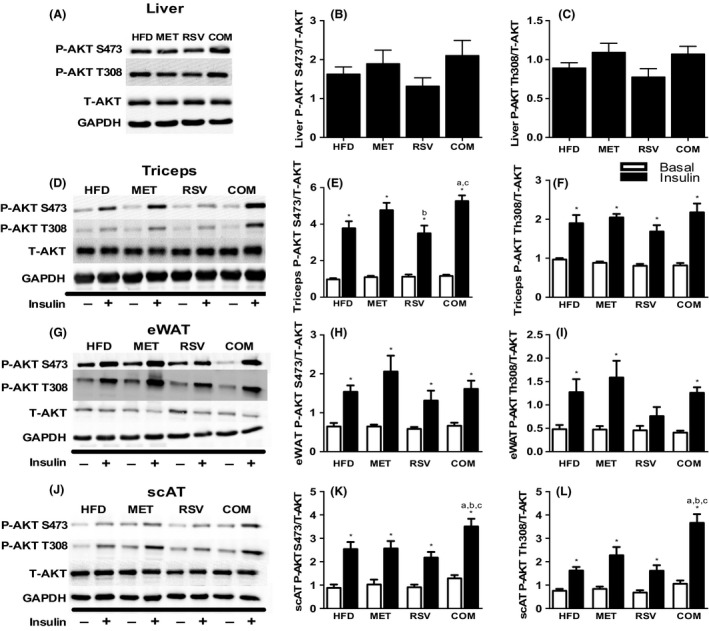
Differences in liver (A–C), triceps (D–F), epididymal (eWAT) (G–I) and subcutaneous (scAT) (J–L) Akt S473 and T308 phosphorylation under basal (□) and insulin stimulated (■) conditions. Representative blots for total and phosphorylated Akt (S473 and T308) in liver (A), triceps (D), eWAT (G), and scAT (J). Data are presented as means ± SEM for 7–11 mice/group. Significance compared to basal within group *P < 0.05. Between‐group significance (*P* < 0.05) is denoted by letters: a different than HFD, b different than MET, c different than RSV and d different than COM.

There were no differences in AKT phosphorylation between groups in the liver (Fig. 4A–C). Insulin significantly increased triceps S473 and T308 AKT phosphorylation in all groups compared to basal (Fig. 4D–F). COM treatment exhibited greater insulin‐stimulated AKT S473 phosphorylation in triceps compared to HFD or RSV alone. Insulin‐induced S473 phosphorylation was greater in triceps muscles from MET compared to RSV‐treated mice (Fig. 4E). No differences were observed in insulin‐induced AKT T308 phosphorylation in triceps between groups (Fig. 4F).

Insulin increased S473 and T308 AKT phosphorylation in eWAT and scAT in all groups compared to basal, with the exception of RSV which did not exhibit increased AKT T308 phosphorylation following insulin‐stimulation in eWAT (Fig. 4G–I). No differences in eWAT AKT phosphorylation were observed between groups (Fig. 4G–I). COM treatment significantly increased insulin‐stimulated S473 and T308 AKT phosphorylation in scAT compared to all other groups (Fig. 4J–L).

### Gluconeogenic enzyme protein content

To determine if changes in gluconeogenic enzymes in the liver could explain the improvements in glucose homeostasis, we measured changes in PEPCK and G6Pase. The protein content of PEPCK and G6Pase was not different in livers from HFD, MET, RSV, or COM treated mice (Figure S1).

### Adiponectin and inflammatory gene expression in adipose tissue

**Figure 5:**
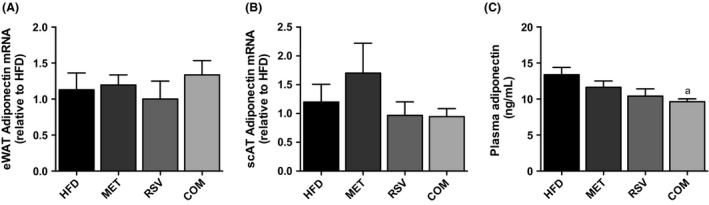
Resveratrol (RSV), metformin (MET), and combined (COM) treatment had no affect on epididymal (eWAT) (A) and subcutaneous (scAT) (B) adiponectin expression, while COM treatment displayed reduced serum adiponectin (C) compared to HFD. Values are presented as mean ± SEM for 6 mice/group in (A–B), and 9–10 mice/group in (C). Significance (*P* < 0.05) is denoted by letters: a different than HFD, b different than MET, c different than RSV and d different than COM.

In order to examine potential mechanisms responsible for the whole‐body effects of COM treatment, we examined adiponectin expression in adipose tissue and plasma, as well as inflammatory gene expression in adipose tissue. No differences between groups were observed in adiponectin expression in eWAT (Fig. 5A) or scAT (Fig. 5B). However, COM exhibited significantly reduced plasma adiponectin compared to HFD (Fig. 5C).

**Figure 6:**
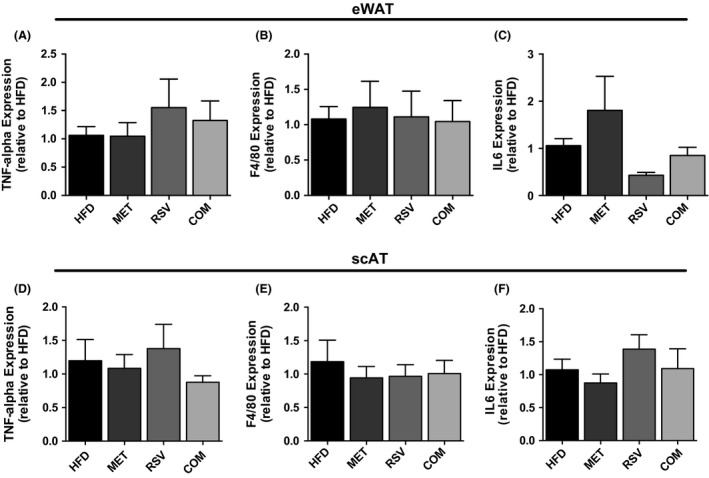
Resveratrol (RSV), metformin (MET), and combined (COM) therapy had no affect on the gene expression of inflammatory markers TNF‐alpha and IL6 or the macrophage marker F4/80 in eWAT (A–C) or scAT (D–F). Values are presented as mean ± SEM for 4–6 mice/group. Significance (*P* < 0.05) is denoted by letters: a different than HFD, b different than MET, c different than RSV and d different than COM.

Adipose tissue inflammation is thought to play a role in the development of insulin resistance (Xu et al. 2003; Lee et al. 2011). As such, we were interested in examining if RSV and/or MET treatment altered markers of adipose tissue inflammation. As shown in Figure 6, no differences were observed in the mRNA expression of TNF‐*α* or interleukin 6 (IL6) in eWAT and scWAT. Moreover, the mRNA expression of F4/80, a marker of macrophage content (Leenen et al. 1994) was not affected by treatment with RSV and MET either alone or in combination in both fat depots. As a further measure of inflammation, we assessed the number of crown‐like structures (CLS) in H&E‐stained scAT sections. This fat depot was analyzed as the most robust changes in insulin‐stimulated AKT phosphorylation occurred in this fat pad. There were no differences in the percentage of adipocytes that were surrounded by CLS between groups (HFD 1.83 ± 0.51, MET 0.93 ± 0.27, RSV 1.07 ± 0.30, COM 0.99 ± 0.31, data are means ± SEM for 5 mice/group).

## Discussion

To the best of our knowledge, this is the first study to compare the individual and combined effects of RSV and MET in treating perturbations in glucose metabolism. In this regard, MET and RSV treatment alone did not significantly improve glucose tolerance, though it should be noted that the glucose AUC was reduced ~20% and 10% in MET‐ and RSV‐treated mice, respectively. However, when given in combination, there was a significant improvement in glucose tolerance. In fact, the observed improvements in glucose tolerance with COM treatment were nearly identical to the sum of RSV and MET improvements administered independently and would suggest an additive effect between these two compounds. Although the dose of RSV used in this study is similar to what has been used in previous rodent‐based investigations (Lagouge et al. 2006), it is far greater than the maximal dose recommended for long‐term clinical use in humans (EFSA Panel on Dietetic Products NaA, 2016). Given the likely limited translatability to humans, the results of the current investigation should be viewed cautiously.

The findings of the current investigation are similar to those of a previous study that found improved whole‐body insulin sensitivity with a combination of MET, RSV, and hydroxymethylbutyrate (HMB) treatment (Bruckbauer and Zemel 2013). However, this study used a genetic model of diabetes, male *db/db* mice (C57BLK6/J‐*lepr* ^*db*^ */lepr* ^*db*^)*,* and the dose of RSV that was used (12.5 mg/kg diet) was much lower than that in the current investigation. Similarly, HMB was given at a comparatively larger dose than both RSV and MET and therefore the results of this particular study are most likely a result of the combined effects of MET and HMB, not RSV.

While MET has consistently been shown to improve glucose homeostasis in conditions of insulin resistance (Stafford and Elasy 2007), the effects of RSV are less clear. For instance, while RSV treatment prevents the development of diet‐induced insulin resistance in rodents (Baur et al. 2006; Lagouge et al. 2006; Beaudoin et al. 2013), RSV supplementation, especially in humans, has marginal effects on improving glucose homeostasis in conditions of pre‐existing insulin resistance and obesity (Timmers et al. 2011; Yoshino et al. 2012; Faghihzadeh et al. 2015). At least at the doses utilized in this study, MET would appear to be more effective than RSV in improving glucose homeostasis. In support of this contention, insulin tolerance was improved in MET but not RSV‐treated mice. Moreover, improvements in glucose tolerance, as measured through reductions in glucose AUC, were ~2 fold greater in MET compared to RSV‐treated mice. Lastly, insulin‐stimulated AKT phosphorylation in eWAT and triceps muscles were reduced in RSV, but not MET‐treated mice when compared to control HFD mice. These findings, taken in context with previous work (Baur et al. 2006; Lagouge et al. 2006; Beaudoin et al. 2013), would suggest that RSV is more effective in preventing the development of insulin resistance than treating it.

The improvements in whole‐body glucose homeostasis following COM treatment were mirrored by increases in insulin‐stimulated AKT phosphorylation in scAT and to a lesser extent triceps muscle. Although MET is not thought to increase adipose tissue insulin action (Ciaraldi et al. 2002), long‐term (13 week) RSV treatment has been reported to increase insulin signaling in this tissue (Svensson et al. 2015). Conversely, treating 3T3‐L1 adipocytes with a high dose of RSV (50 μmol/L) has been shown to induce insulin resistance (Wang et al. 2011). In this study, we found that the insulin‐induced phosphorylation of AKT on threonine 308 was markedly impaired in eWAT from RSV‐treated mice. As both MET and RSV did not improve, and in some cases reduced, insulin signaling in adipose tissue, enhanced insulin‐stimulated AKT phosphorylation with COM treatment likely speaks to a synergistic effect of these two compounds in improving scAT insulin signaling.

The association between increases in scAT and triceps insulin signaling and improvements in glucose tolerance in COM‐treated mice suggests a role for enhanced insulin signaling in these tissues being involved in improvements in whole‐body glucose homeostasis. Consistent with the potential importance of scAT in mediating whole‐body improvements in glucose homeostasis, a recent study has reported similar associations between key proteins involved in insulin signaling (AKT, GLUT4) in subcutaneous adipose tissue and indices of insulin sensitivity in obese subjects following diet‐induced weight loss (Fritzen et al. 2015).

While we have demonstrated a clear enhancement of insulin signaling in scAT, this does not appear to be explained by alterations in markers of inflammation, or through increases in the expression, or circulating levels, of adiponectin. Moreover, the effects of RSV and MET in combination are not secondary to differences in body weight or adiposity (i.e., fat cell size and adipose tissue weight). Future work aimed at identifying the cellular events leading to enhanced insulin signaling in adipose tissue with dual RSV and MET therapy is needed and could help in identifying new targets to improve glucose homeostasis.

In summary, this study has been the first to compare the individual and combined effects of MET and RSV in the treatment of insulin resistance. MET appears to be more effective in treating insulin resistance than RSV, at least at the concentrations utilized. Moreover, we have provided evidence of potential beneficial effects of combined RSV and MET treatment on indices of whole‐body glucose homeostasis that might be explained, at least in part, through increases in insulin signaling in adipose tissue and muscle.

## Conflicts of Interest

None declared.

## Supporting information




**Figure S1.** RSV, MET and COM therapy had no affect on the protein content of PEPCK and G6Pase in the liver. Values are presented as mean ± SEM for 8–10 mice/group.

## References

[phy212877-bib-0001] Baur, J. A. , K. J. Pearson , N. L. Price , H. A. Jamieson , C. Lerin , A. Kalra , et al. 2006 Resveratrol improves health and survival of mice on a high‐calorie diet. Nature 444:337–342. 17086191 10.1038/nature05354 PMC4990206

[phy212877-bib-0002] Beaudoin, M. S. , L. A. Snook , A. M. Arkell , J. A. Simpson , G. P. Holloway , and D. C. Wright . 2013 Resveratrol supplementation improves white adipose tissue function in a depot‐specific manner in Zucker diabetic fatty rats. Am. J. Physiol. Regul. Integr. Comp. Physiol. 305:R542–R551. 23824959 10.1152/ajpregu.00200.2013

[phy212877-bib-0003] Bruce, C. R. , V. A. Mertz , G. J. Heigenhauser , and D. J. Dyck . 2005 The stimulatory effect of globular adiponectin on insulin‐stimulated glucose uptake and fatty acid oxidation is impaired in skeletal muscle from obese subjects. Diabetes 54:3154–3160. 16249439 10.2337/diabetes.54.11.3154

[phy212877-bib-0004] Bruckbauer, A. , and M. B. Zemel . 2013 Synergistic effects of metformin, resveratrol, and hydroxymethylbutyrate on insulin sensitivity. Diabetes Metab. Syndr. Obes. 6:93–102. 23430507 10.2147/DMSO.S40840 PMC3575126

[phy212877-bib-0005] Ciaraldi, T. P. , A. P. Kong , N. V. Chu , D. D. Kim , S. Baxi , M. Loviscach , et al. 2002 Regulation of glucose transport and insulin signaling by troglitazone or metformin in adipose tissue of type 2 diabetic subjects. Diabetes 51:30–36. 11756319 10.2337/diabetes.51.1.30

[phy212877-bib-0006] DeFronzo, R. A. 1999 Pharmacologic therapy for type 2 diabetes mellitus. Ann. Intern. Med. 131:281–303. 10454950 10.7326/0003-4819-131-4-199908170-00008

[phy212877-bib-0007] EFSA Panel on Dietetic Products NaA . 2016 Safety of synthetic trans‐resveratrol as a novel food pursuant to Regulation (EC) No 258/97. EFSA J. 14:4368.

[phy212877-bib-0008] Faghihzadeh, F. , P. Adibi , and A. Hekmatdoost . 2015 The effects of resveratrol supplementation on cardiovascular risk factors in patients with non‐alcoholic fatty liver disease: a randomised, double‐blind, placebo‐controlled study. Br. J. Nutr. 114:796–803. 26234526 10.1017/S0007114515002433

[phy212877-bib-0009] Fritzen, A. M. , A. M. Lundsgaard , A. B. Jordy , S. K. Poulsen , S. Stender , H. Pilegaard , et al. 2015 New nordic diet‐induced weight loss is accompanied by changes in metabolism and AMPK signaling in adipose tissue. J. Clin. Endocrinol. Metab. 100:3509–3519. 26126206 10.1210/jc.2015-2079

[phy212877-bib-0010] Fujita, H. , H. Fujishima , T. Morii , J. Koshimura , T. Narita , M. Kakei , et al. 2002 Effect of metformin on adipose tissue resistin expression in db/db mice. Biochem. Biophys. Res. Commun. 298:345–349. 12413946 10.1016/s0006-291x(02)02464-6

[phy212877-bib-0011] Hou, M. , N. Venier , L. Sugar , M. Musquera , M. Pollak , A. Kiss , et al. 2010 Protective effect of metformin in CD1 mice placed on a high carbohydrate‐high fat diet. Biochem. Biophys. Res. Commun. 397:537–542. 20573602 10.1016/j.bbrc.2010.05.152

[phy212877-bib-0012] Hundal, R. S. , M. Krssak , S. Dufour , D. Laurent , V. Lebon , V. Chandramouli , et al. 2000 Mechanism by which metformin reduces glucose production in type 2 diabetes. Diabetes 49:2063–2069. 11118008 10.2337/diabetes.49.12.2063 PMC2995498

[phy212877-bib-0013] Inzucchi, S. E. , D. G. Maggs , G. R. Spollett , S. L. Page , F. S. Rife , V. Walton , et al. 1998 Efficacy and metabolic effects of metformin and troglitazone in type II diabetes mellitus. N. Engl. J. Med. 338:867–872. 9516221 10.1056/NEJM199803263381303

[phy212877-bib-0014] Koh, E. H. , J. Y. Park , H. S. Park , M. J. Jeon , J. W. Ryu , M. Kim , et al. 2007 Essential role of mitochondrial function in adiponectin synthesis in adipocytes. Diabetes 56:2973–2981. 17827403 10.2337/db07-0510

[phy212877-bib-0015] Kristensen, J. M. , S. Larsen , J. W. Helge , F. Dela , and J. F. Wojtaszewski . 2013 Two weeks of metformin treatment enhances mitochondrial respiration in skeletal muscle of AMPK kinase dead but not wild type mice. PLoS ONE 8:e53533. 23341947 10.1371/journal.pone.0053533 PMC3544921

[phy212877-bib-0016] Lagouge, M. , C. Argmann , Z. Gerhart‐Hines , H. Meziane , C. Lerin , F. Daussin , et al. 2006 Resveratrol improves mitochondrial function and protects against metabolic disease by activating SIRT1 and PGC‐1alpha. Cell 127:1109–1122. 17112576 10.1016/j.cell.2006.11.013

[phy212877-bib-0017] Lee, Y. S. , P. Li , J. Y. Huh , I. J. Hwang , M. Lu , J. I. Kim , et al. 2011 Inflammation is necessary for long‐term but not short‐term high‐fat diet‐induced insulin resistance. Diabetes 60:2474–2483. 21911747 10.2337/db11-0194 PMC3178297

[phy212877-bib-0018] Leenen, P. J. , M. F. de Bruijn , J. S. Voerman , P. A. Campbell , and W. van Ewijk . 1994 Markers of mouse macrophage development detected by monoclonal antibodies. J. Immunol. Methods 174:5–19. 8083537 10.1016/0022-1759(94)90005-1

[phy212877-bib-0019] Livak, K. J. , and T. D. Schmittgen . 2001 Analysis of relative gene expression data using real‐time quantitative PCR and the 2(‐Delta Delta C(T)) Method. Methods 25:402–408. 11846609 10.1006/meth.2001.1262

[phy212877-bib-0020] Luna, V. , L. Casauban , M. P. Sajan , J. Gomez‐Daspet , J. L. Powe , A. Miura , et al. 2006 Metformin improves atypical protein kinase C activation by insulin and phosphatidylinositol‐3,4,5‐(PO4)3 in muscle of diabetic subjects. Diabetologia 49:375–382. 16395615 10.1007/s00125-005-0112-4

[phy212877-bib-0021] MacPherson, R. E. , J. S. Huber , S. Frendo‐Cumbo , J. A. Simpson , and D. C. Wright . 2015 Adipose tissue insulin action and IL‐6 signaling following exercise in obese mice. Med. Sci. Sports Exerc. 2034–2042. 10.1249/MSS.0000000000000660 25785928

[phy212877-bib-0022] MacPherson, R. E. , D. Gamu , S. Frendo‐Cumbo , L. Castellani , F. Kwon , A. R. Tupling , et al. 2016 Sarcolipin knockout mice fed a high fat diet exhibit altered indices of adipose tissue inflammation and remodelling. Obesity (Silver Spring) 24:1499–1505. 27345961 10.1002/oby.21521

[phy212877-bib-0023] Mathers, C. D. , and D. Loncar . 2006 Projections of global mortality and burden of disease from 2002 to 2030. PLoS Med. 3:e442. 17132052 10.1371/journal.pmed.0030442 PMC1664601

[phy212877-bib-0024] Miyazaki, Y. , A. Mahankali , M. Matsuda , L. Glass , S. Mahankali , E. Ferrannini , et al. 2001 Improved glycemic control and enhanced insulin sensitivity in type 2 diabetic subjects treated with pioglitazone. Diabetes Care 24:710–719. 11315836 10.2337/diacare.24.4.710

[phy212877-bib-0025] Miyazaki, Y. , A. Mahankali , M. Matsuda , S. Mahankali , J. Hardies , K. Cusi , et al. 2002 Effect of pioglitazone on abdominal fat distribution and insulin sensitivity in type 2 diabetic patients. J. Clin. Endocrinol. Metab. 87:2784–2791. 12050251 10.1210/jcem.87.6.8567

[phy212877-bib-0026] Musi, N. , M. F. Hirshman , J. Nygren , M. Svanfeldt , P. Bavenholm , O. Rooyackers , et al. 2002 Metformin increases AMP‐activated protein kinase activity in skeletal muscle of subjects with type 2 diabetes. Diabetes 51:2074–2081. 12086935 10.2337/diabetes.51.7.2074

[phy212877-bib-0027] Nawrocki, A. R. , M. W. Rajala , E. Tomas , U. B. Pajvani , A. K. Saha , M. E. Trumbauer , et al. 2006 Mice lacking adiponectin show decreased hepatic insulin sensitivity and reduced responsiveness to peroxisome proliferator‐activated receptor gamma agonists. J. Biol. Chem. 281:2654–2660. 16326714 10.1074/jbc.M505311200

[phy212877-bib-0028] Nissen, S. E. , and K. Wolski . 2007 Effect of rosiglitazone on the risk of myocardial infarction and death from cardiovascular causes. N. Engl. J. Med. 356:2457–2471. 17517853 10.1056/NEJMoa072761

[phy212877-bib-0029] Raj, P. , X. L. Louis , S. J. Thandapilly , A. Movahed , S. Zieroth , and T. Netticadan . 2014 Potential of resveratrol in the treatment of heart failure. Life Sci. 95:63–71. 24361400 10.1016/j.lfs.2013.12.011

[phy212877-bib-0030] Smith, P. K. , R. I. Krohn , G. T. Hermanson , A. K. Mallia , F. H. Gartner , M. D. Provenzano , et al. 1985 Measurement of protein using bicinchoninic acid. Anal. Biochem. 150:76–85. 3843705 10.1016/0003-2697(85)90442-7

[phy212877-bib-0031] Stafford, J. M. , and T. Elasy . 2007 Treatment update: thiazolidinediones in combination with metformin for the treatment of type 2 diabetes. Vasc. Health Risk Manag. 3:503–510. 17969380 PMC2291335

[phy212877-bib-0032] Sung, M. M. , and J. R. Dyck . 2015 Therapeutic potential of resveratrol in heart failure. Ann. N. Y. Acad. Sci. 1348:32–45. 26205211 10.1111/nyas.12839

[phy212877-bib-0033] Svensson, K. , S. Schnyder , V. Albert , B. Cardel , L. Quagliata , L. M. Terracciano , et al. 2015 Resveratrol and SRT1720 elicit differential effects in metabolic organs and modulate systemic parameters independently of skeletal muscle peroxisome proliferator‐activated receptor gamma co‐activator 1alpha (PGC‐1alpha). J. Biol. Chem. 290:16059–16076. 25987562 10.1074/jbc.M114.590653 PMC4481209

[phy212877-bib-0034] Timmers, S. , E. Konings , L. Bilet , R. H. Houtkooper , T. van de Weijer , G. H. Goossens , et al. 2011 Calorie restriction‐like effects of 30 days of resveratrol supplementation on energy metabolism and metabolic profile in obese humans. Cell Metab. 14:612–622. 22055504 10.1016/j.cmet.2011.10.002 PMC3880862

[phy212877-bib-0035] Turner, R. M. , C. S. Kwok , C. Chen‐Turner , C. A. Maduakor , S. Singh , and Y. K. Loke . 2014 Thiazolidinediones and associated risk of Bladder Cancer: a Systematic Review and Meta‐analysis. Br. J. Clin. Pharmacol. 78:258–273. 24325197 10.1111/bcp.12306 PMC4137819

[phy212877-bib-0036] Wang, A. , M. Liu , X. Liu , L. Q. Dong , R. D. Glickman , T. J. Slaga , et al. 2011 Up‐regulation of adiponectin by resveratrol: the essential roles of the Akt/FOXO1 and AMP‐activated protein kinase signaling pathways and DsbA‐L. J. Biol. Chem. 286:60–66. 20980258 10.1074/jbc.M110.188144 PMC3013020

[phy212877-bib-0037] Wu, M. L. , H. Li , L. J. Yu , X. Y. Chen , Q. Y. Kong , X. Song , et al. 2014 Short‐term resveratrol exposure causes in vitro and in vivo growth inhibition and apoptosis of bladder cancer cells. PLoS ONE 9:e89806. 24587049 10.1371/journal.pone.0089806 PMC3934942

[phy212877-bib-0038] Xu, H. , G. T. Barnes , Q. Yang , G. Tan , D. Yang , C. J. Chou , et al. 2003 Chronic inflammation in fat plays a crucial role in the development of obesity‐related insulin resistance. J. Clin. Invest. 112:1821–1830. 14679177 10.1172/JCI19451 PMC296998

[phy212877-bib-0039] Yamauchi, T. , J. Kamon , Y. Minokoshi , Y. Ito , H. Waki , S. Uchida , et al. 2002 Adiponectin stimulates glucose utilization and fatty‐acid oxidation by activating AMP‐activated protein kinase. Nat. Med. 8:1288–1295. 12368907 10.1038/nm788

[phy212877-bib-0040] Yoshino, J. , C. Conte , L. Fontana , B. Mittendorfer , S. Imai , K. B. Schechtman , et al. 2012 Resveratrol supplementation does not improve metabolic function in nonobese women with normal glucose tolerance. Cell Metab. 16:658–664. 23102619 10.1016/j.cmet.2012.09.015 PMC3496026

