# Supplementary material for: Beneficial effects of combined resveratrol and metformin therapy in treating diet‐induced insulin resistance
Source: Physiol Rep. 2016 Aug 1;4(15):e12877. doi: 10.14814/phy2.12877 (PMC4985545; doi:10.14814/phy2.12877)
Supplement: Supplementary file 1 — Figure S1. RSV, MET and COM therapy had no affect on the protein content of PEPCK and G6Pase in the liver. Values are presented as mean ± SEM for 8–10 mice/group. [file PHY2-4-e12877-s001.docx]

**Supplemental Figure 1:** RSV, MET and COM therapy had no affect on the protein content of PEPCK and G6Pase in the liver. Values are presented as mean ± SEM for 8-10 mice/group.
